# A Chromosomal Deletion and New Frameshift Mutation Cause ARSACS in an African-American

**DOI:** 10.3389/fneur.2018.00956

**Published:** 2018-11-15

**Authors:** Sean C. Dougherty, Amy Harper, Hind Al Saif, Gregory Vorona, Scott R. Haines

**Affiliations:** ^1^Department of Neurology, Virginia Commonwealth University, Richmond, VA, United States; ^2^Department of Human and Molecular Genetics, Virginia Commonwealth University, Richmond, VA, United States; ^3^Department of Radiology, Virginia Commonwealth University, Richmond, VA, United States

**Keywords:** ARSACS, ataxia, Charlevoix-Saguenay, cerebellum, neurogenetics, polyneuropathy, spasticity, retina

## Abstract

Autosomal Recessive Spastic Ataxia of Charlevoix-Saguenay (ARSACS) is a rare, progressive, neurodegenerative disease characterized by ataxia, spasticity and polyneuropathy. First described in the French-Canadian population of Quebec in 1978, ARSACS has since been identified in multiple patients worldwide. In this clinical case report, we describe the evaluation of an 11-years-old African-American male who presented to neuromuscular clinic for assessment of a gait abnormality. He had a history of gross motor delay since early childhood, frequent falls and a below average IQ. Chromosomal microarray revealed a 1.422 megabase loss in the 13q12.12 region, which includes the *SACS* gene. Next Generation Sequencing then showed a novel, predicted to be pathogenic missense mutation (c.11824dup) of this gene. His clinical presentation and neurological imaging further confirmed the diagnosis of ARSACS. To our knowledge, this is the first reported case of this disease in the African-American population of the United States. This case report further highlights the growing trend of identifying genetic diseases previously restricted to single, ethnically isolated regions in many different ethnic groups worldwide.

## Background

Autosomal Recessive Spastic Ataxia of Charlevoix-Saguenay (ARSACS) is a rare neurodegenerative condition initially identified in the French-Canadian population in 1978 (1). Secondary to a mutation of the *SACS* gene on chromosome 13q, this disease is characterized by cerebellar ataxia, spasticity, polyneuropathy, nystagmus and retinal changes (2). It is inherited in an autosomal recessive manner and typically manifests in early childhood as difficulty with ambulation and gait unsteadiness. ARSACS is slowly progressive, eventually leading to loss of ambulation around age 40 (range 17–58) and death around age 50 (range 21–72) (3).

Originally characterized in two regions of northeastern Quebec previously settled by individuals of French descent (4), ARSACS has since been identified in multiple patients worldwide. Whereas, most of the affected patients in French-Canada had the same single nucleotide deletion in the exon of the *SACS* gene, new missense mutations have been identified in individuals from Tunisia, Turkey, Italy, Japan and multiple countries in Europe (5–11).

The worldwide incidence of ARSACS is unknown, though it is thought to be underdiagnosed. Within the Saguenay-Lac-St-Jean region of Quebec, however, the estimated incidence is between 1 in 1,500 to 2,000 individuals. The estimated carrier frequency of *SACS* pathogenic variants is 1 in 21 individuals based on population data accumulated between 1941 and 1985 (4, 12, 13).

In this clinical case report, we detail the first published case of ARSACS in an African-American patient in the United States, highlighting the clinical presentation, neurological imaging and genetic analysis involved in diagnosis. Furthermore, we identified a novel, predicted to be pathogenic missense mutation in the *SACS* gene (c.11824dup) resulting in the ARSACS phenotype.

## Case presentation

**Figure 1:**
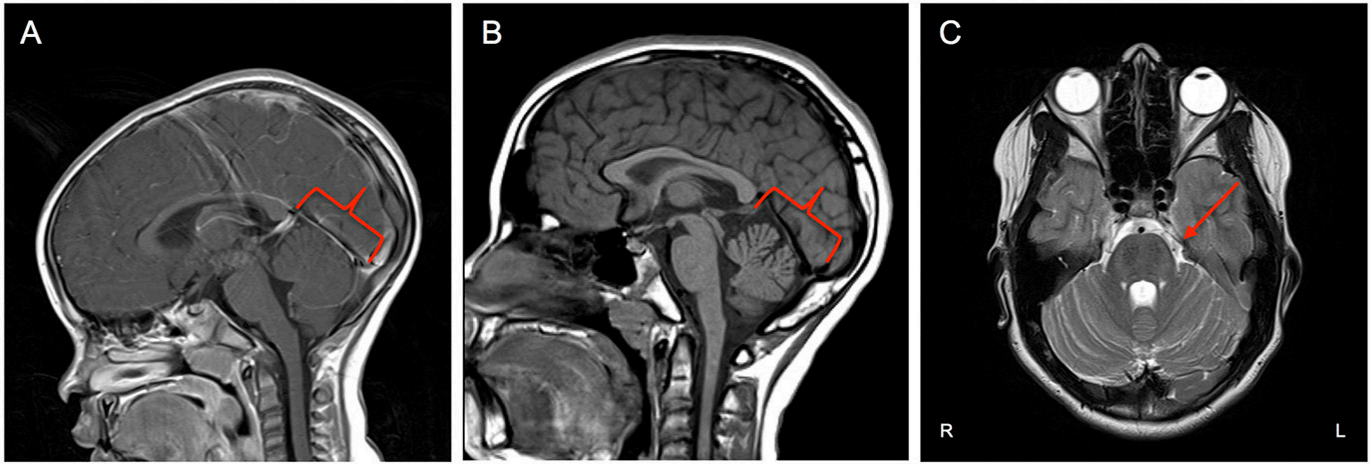
**(A)** Midsagittal MRI at age 3 showing no cerebellar changes at that time. **(B)** Midsagittal MRI at age 11 showing evidence of asymmetric volume loss within the superior and middle components of the cerebellar vermis with preserved volume of the inferior component (red outline). **(C)** Axial plane MRI exhibiting faint linear/T2 signal hypointensities within the Pons (red arrow).

An 11 years-old African-American male presented to pediatric neuromuscular clinic for evaluation of gait abnormality. He had a history of gross motor delay since age 3 and was enrolled in both physical and occupational therapy. His mother noted frequent falls, particularly when running or getting onto the school bus, as well as chronic headaches and blurry vision. Previously performed neuropsychological testing showed a below average IQ and an MRI of the brain at age 3 was normal (Figure 1A). An MRI of the lumbosacral spine from age 5 was also reportedly normal. Prior laboratory evaluation, including CPK, ANA and Acetylcholine receptor antibodies, was normal. He had never had a formal eye exam. Family history was significant for a maternal great grandfather who required leg braces starting at age 6 (with removal around age 10), who also reportedly had a similar posture as the patient. There was also a family history of non-specific visual symptoms in multiple family members on his mother's side as well as an older sibling who previously had seizures. He was not of French-Canadian descent, was the child of a non-consanguineous conception and had two half-brothers and one half-sister, all of whom were alive and well aside from the aforementioned seizures. Both of his parents and all of his grandparents were African-American as well.

**Figure 2:**
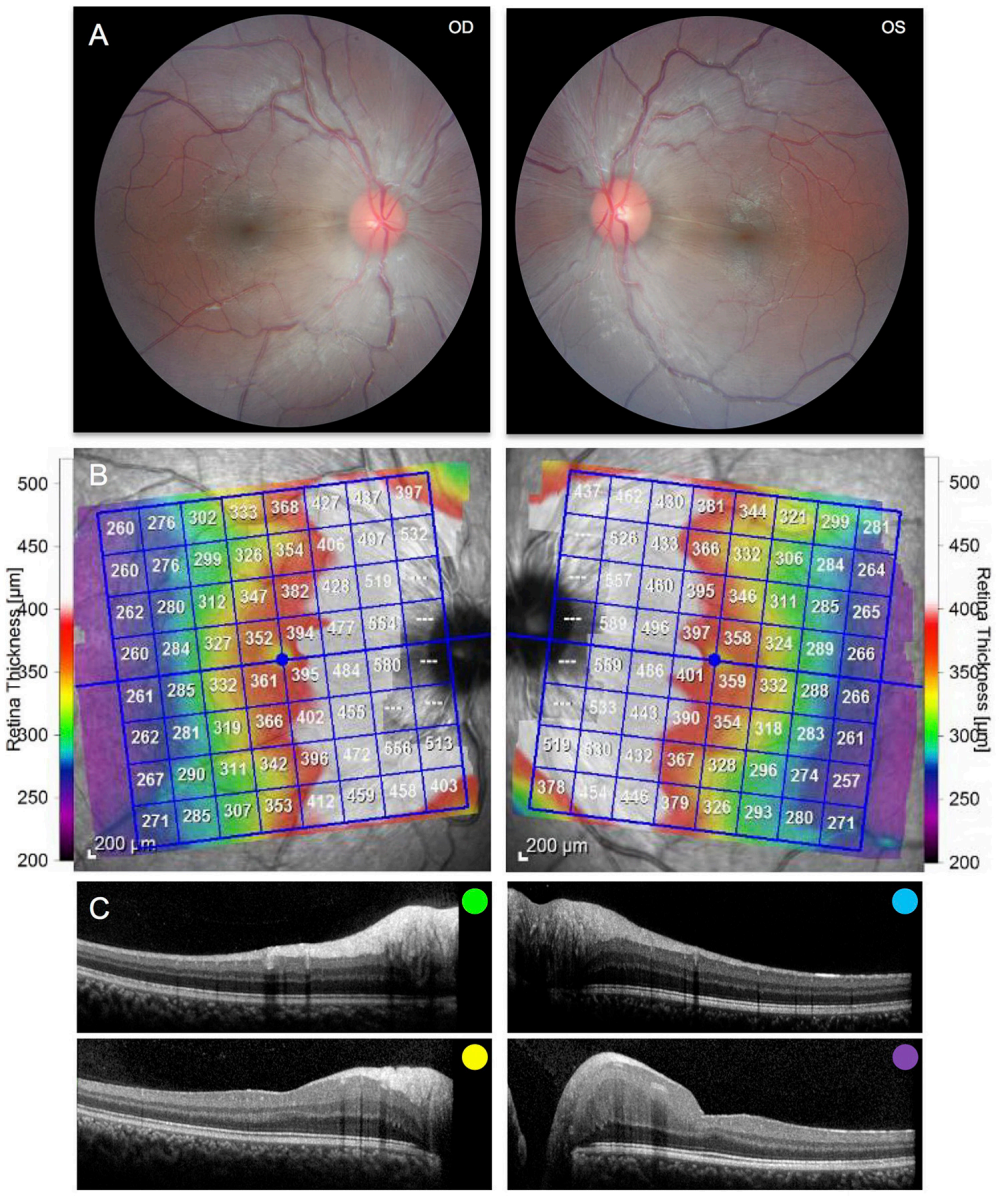
**(A)** Fundus photography of the right and left eyes demonstrating peripapillary retinal nerve fiber hypertrophy and white striations emanating adjacent to the optic nerve. **(B)** Posterior pole Optical Coherence Tomography demonstrating hypertrophy of the retinal nerve fiber layer and other inner retinal layers. **(C)** Selected cross sections of the right and left eye demonstrating hypertrophy of the retinal nerve fiber layer and other inner retinal layers. Colors correspond to the retinal layers depicted above.

On examination, he had gaze-evoked nystagmus and saccadic dysmetria with undershoot saccades in all directions, prominent white striations emanating adjacent to the optic nerve withdistinct optic disc margins on fundoscopy (Figure 2A), and symmetric pupils with no afferent defect. Subtle choreiform movements were noted in the fingers with outstretched hands and end point tremor on finger to nose testing was present. In the lower extremities, there was increased tone of spastic nature. Patellar reflexes were brisk and ankle jerks were decreased with associated weakness. Cross adduction was present. His gait was wide based and staggering; he was unable to perform tandem gait testing. Spasticity with mild scissoring was apparent with casual gait testing.

Brain MRI revealed asymmetric volume loss within the superior and middle aspects of the cerebellar vermis relative to the inferior aspect (Figure 1B) and faint linear T2 signal hypointensities within the pons (Figure 1C), findings that have been shown to be associated with ARSACS (14, 15). Fundoscopic photography and Optical Coherence Tomography both demonstrated hypertrophy of the retinal nerve fiber layer and other retinal layers (Figures 2B,C), findings also observed in ARSACS (16). Electromyography performed was abnormal for both sensory and motor components in multiple nerves tested, providing electrophysiological evidence for a sensory greater than motor axonal polyneuropathy.

**Figure 3:**
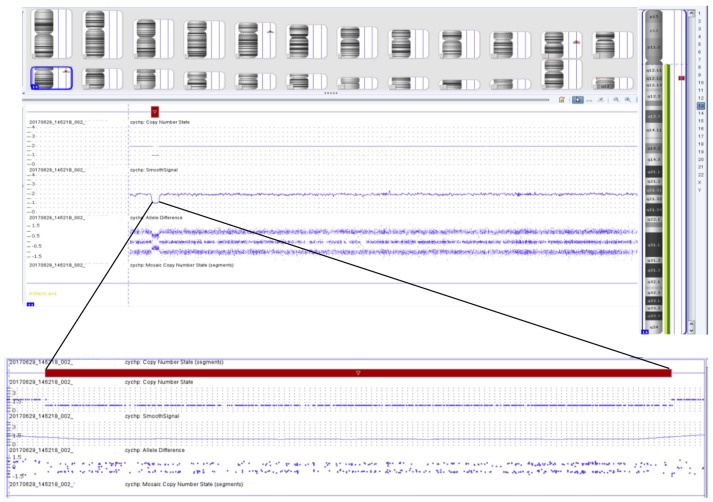
Chromosomal microarray showing 1.422 megabase loss in the 13q12.12 region containing 14 different genes, including the *SACS* gene.

Initial chromosome microarray then showed a 1.422 megabase loss in the 13q12.12 region (Figure 3), of which there are 14 genes present. Three of these genes have autosomal recessive genetic conditions associated with them, including Limb-girdle muscular dystrophy type 2C (*SGCG* gene), ARSACS (*SACS* gene), and Combined oxidative phosphorylation deficiency 31 (*MIPEP* gene). Subsequent Next Generation Sequencing of these genes revealed that the patient had hemizygous, likely pathogenic variant in the *SACS* gene (c.11824dup), which was predicted to result in a frameshift mutation with premature protein termination (p.Met3942Asnfs^*^4). Taken together, these clinical and genetic findings are consistent with the diagnosis of ARSACS.

Current treatment is focused on symptomatic management and improvement of daily life. Gait stabilization with the wearing of ankle foot orthoses has been observed and he has been receiving scheduled botox injections to the bilateral hamstrings every 90 days with improvement in flexibility.

## Discussion

With the implementation of targeted high throughput sequencing and identification of novel genetic mutations, ARSACS, a disease previously reported primarily in the French-Canadian population of Quebec, is now being identified worldwide (17). Here, we have described the clinical presentation, diagnostic evaluation and management of the first reported case of ARSACS in the African-American population. Furthermore, we identified a previously unreported mutation in the *SACS* gene, designated c. 11824dup.

A number of different mutations have been shown to cause the ARSACS phenotype (18–20), which is thought to result from loss of function of the encoded protein Sacsin. The exact role of this large, 520-kDa cytoplasmic protein remains unknown, though it has been proposed to have roles in mitochondrial function (21), protein chaperoning (22), and the ubiquitin-proteasome system (23). Further studies have shown that Sacsin knockout results in increased reactive oxygen species production (24) and alterations in synaptic input and intrinsic firing in cerebellar Purkinje cells (25).

The observed genetics in this patient are atypical in that he is a compound heterozygote, wherein full chromosomal deletion of one of the *SACS* genes combined with the observed frameshift mutation (hemizygous) resulted in the ARSACS phenotype. A hemizygous presentation of this disease has previously been described in the Belgian population (26). These two cases are unique in that they involve gross chromosomal deletion of the *SACS* gene, whereas all previously reported mutations in patients with ARSACS involved either point mutations or missense mutations in this gene. Moderate perceptive hearing loss was described as the only feature by which the Belgian patient with ARSACS differed from other cases of the disease, however this feature was not present in our patient.

Despite a family history of gait abnormalities on his mother's side, we cannot comment specifically on the nature of this patient's inheritance without formal genetic testing of his parents, which has not been performed at this time. *De novo* or inherited deletion of the *SACS* gene from one parent combined with another *de novo* or inherited likely pathogenic variant from the other parent would be a theory of inheritance compatible with the history and known genetics of this patient.

## Concluding remarks

In sum, a high index of clinical suspicion combined with appropriate genetic testing should allow for continued identification of the ARSACS phenotype, a disease whose worldwide prevalence will undoubtedly continue to rise as further novel mutations are characterized.

## Ethics statement

All clinical data in this case report was collected with the consent of the patient and his mother. A written informed consent was obtained from the patient and his parents for the participation in the study and the publication of this report. The case report is exempt from institutional review board approval.

## Author contributions

SD wrote the manuscript under the mentorship of SH. AH acquired and interpreted clinical data and aided in diagnosis. HA interpreted genetic data and provided commentary. GV interpreted radiologic images and provided commentary. SH acquired ophthalmologic photographs and clinical data, interpreted data and supervised case report formulation.

### Conflict of interest statement

The authors declare that the research was conducted in the absence of any commercial or financial relationships that could be construed as a potential conflict of interest.

## Abbreviations

ARSACS: Autosomal Recessive Spastic Ataxia of Charlevoix-Saguenay.
